# Supplementary material for: Combining Ability Analysis of Yield-Related Traits of Two Elite Rice Restorer Lines in Chinese Hybrid Rice
Source: Int J Mol Sci. 2023 Aug 3;24(15):12395. doi: 10.3390/ijms241512395 (PMC10418745; doi:10.3390/ijms241512395)
Supplement: Supplementary file 1 [file ijms-24-12395-s001.zip › Figure S1.pdf]

International Journal of
Molecular Sciences

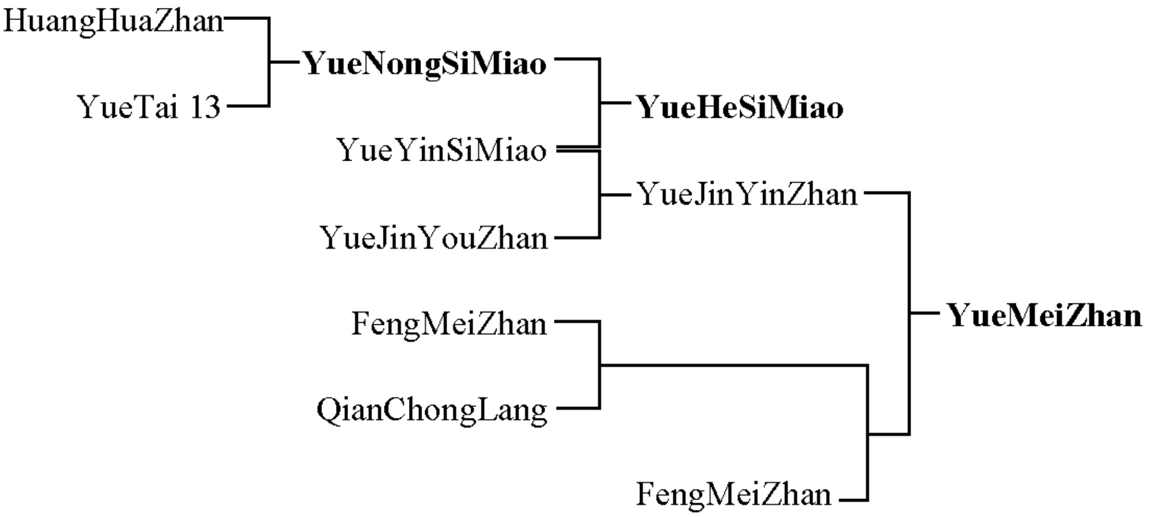

Figure S1. The pedigree details of the parental lines Yuenongsimiao, Yuehesimiao, and Yuemeizhan.
